# Comparison of long-read sequencing technologies in interrogating bacteria and fly genomes

**DOI:** 10.1093/g3journal/jkab083

**Published:** 2021-04-19

**Authors:** Eric S Tvedte, Mark Gasser, Benjamin C Sparklin, Jane Michalski, Carl E Hjelmen, J Spencer Johnston, Xuechu Zhao, Robin Bromley, Luke J Tallon, Lisa Sadzewicz, David A Rasko, Julie C Dunning Hotopp

**Affiliations:** 1 Institute for Genome Sciences, University of Maryland School of Medicine, Baltimore, MD 21201, USA; 2 Department of Microbiology and Immunology, University of Maryland School of Medicine, Baltimore, MD 21201, USA; 3 Department of Biology, Texas A&M University, College Station, TX 77843, USA; 4 Department of Entomology, Texas A&M University, College Station, TX 77843, USA; 5 Greenebaum Cancer Center, University of Maryland School of Medicine, Baltimore, MD 21201, USA

**Keywords:** genomics, bacterial genomics, fly genomics, *Drosophila ananassae*, sequencing

## Abstract

The newest generation of DNA sequencing technology is highlighted by the ability to generate sequence reads hundreds of kilobases in length. Pacific Biosciences (PacBio) and Oxford Nanopore Technologies (ONT) have pioneered competitive long read platforms, with more recent work focused on improving sequencing throughput and per-base accuracy. We used whole-genome sequencing data produced by three PacBio protocols (Sequel II CLR, Sequel II HiFi, RS II) and two ONT protocols (Rapid Sequencing and Ligation Sequencing) to compare assemblies of the bacteria *Escherichia coli* and the fruit fly *Drosophila ananassae*. In both organisms tested, Sequel II assemblies had the highest consensus accuracy, even after accounting for differences in sequencing throughput. ONT and PacBio CLR had the longest reads sequenced compared to PacBio RS II and HiFi, and genome contiguity was highest when assembling these datasets. ONT Rapid Sequencing libraries had the fewest chimeric reads in addition to superior quantification of *E. coli* plasmids versus ligation-based libraries. The quality of assemblies can be enhanced by adopting hybrid approaches using Illumina libraries for bacterial genome assembly or polishing eukaryotic genome assemblies, and an ONT-Illumina hybrid approach would be more cost-effective for many users. Genome-wide DNA methylation could be detected using both technologies, however ONT libraries enabled the identification of a broader range of known *E. coli* methyltransferase recognition motifs in addition to undocumented *D. ananassae* motifs. The ideal choice of long read technology may depend on several factors including the question or hypothesis under examination. No single technology outperformed others in all metrics examined.

## Introduction

Long-read sequencing technologies are widely used to generate both new and improved genome assemblies. Although short read sequencing is cost-effective and highly accurate, the presence of low complexity regions in a genome hinders the resolution of contiguous regions using short reads alone. As specialized tools have been developed to process long-read sequencing data (Amarasinghe *et al.* 2020), complete bacterial genomes are routinely produced with modest coverage (Koren and Phillippy 2015; Wick and Holt 2019) and the near entirety of eukaryotic chromosomes have been resolved in single contiguous sequences (Kolmogorov *et al.* 2018; Adams *et al.* 2020; Logsdon *et al.* 2020; Michael and VanBuren 2020).

The industry leaders in long-read sequencing technologies are Pacific Biosciences (PacBio) and Oxford Nanopore Technologies (ONT). Since the release of the PacBio RS sequencer in 2011 and the ONT MinION sequencer in 2014, improvements in sequencing chemistries and new sequencing platforms have continued to produce longer sequences and higher sequencing throughput, thus decreasing per-base sequencing costs (van Dijk *et al.* 2018). Most recently, the PacBio Sequel II system advertises the highest throughput out of any of its sequencing platforms and includes two distinct single molecule real-time (SMRT) sequencing modes: continuous long-read sequencing (CLR) for ultralong reads and circular consensus sequencing (CCS/HiFi) for highly-accurate consensus reads.

Beyond improving genome assemblies, PacBio and ONT sequencing can be used as an alternative to bisulfite sequencing to detect genome-wide DNA methylation. DNA methylation is found across the tree of life and is associated with a wide range of biological functions, including protection of host DNA against endonuclease cleavage, DNA replication, and gene expression (Sánchez-Romero *et al.* 2015). DNA modification events are detected as measurements of DNA polymerase kinetics in PacBio SMRT sequencing (Clark *et al.* 2012; Lee *et al.* 2015; Payelleville *et al.* 2018) and as changes in the ionic current signal in the ONT nanopore (Rand *et al.* 2017; Simpson *et al.* 2017).

Although long reads can be useful in overcoming potential pitfalls of assembling with short read data alone, there are notable disadvantages of long-read sequencing data. The error rate for single pass sequencing is >10% for both PacBio CLR and ONT sequencers (Jain *et al.* 2017; Ardui *et al.* 2018; Wick *et al.* 2019). Methods have been developed to address these high error rates, such as the use of error correction before or after the assembly to achieve a highly accurate consensus sequence (Sedlazeck *et al.* 2018). The Sequel II HiFi sequencing mode also addresses this issue as >99% accurate consensus reads are produced from multiple passes of a single template molecule during the sequencing run (Wenger *et al.* 2019) and new assembly algorithms are utilizing the increased accuracy of HiFi reads to construct high quality genome sequences (Nurk *et al.* 2020). In addition to the high read error rates of some workflows, DNA sequences that originate from two distinct parent sequences (*i.e.*, chimeric reads) can also hinder the assembly process, although this is also a problem with all prior sequencing platforms. Chimeric reads have been reported in PacBio (Fichot and Norman 2013) and ONT sequencing (White *et al.* 2017), and preparations involving ligation and/or PCR amplification steps are likely to generate such artefacts. Lastly, PacBio and ONT sequencing libraries are more costly to generate than short read libraries (Rhoads and Au 2015).

Here, we investigate the quality of long-read sequencing data produced using five methods: PacBio RS II, PacBio Sequel II CLR, PacBio Sequel II HiFi, ONT Rapid Sequencing Kit (ONT RAPID), and ONT Ligation Sequencing Kit (ONT LIG). We also sequenced Illumina libraries for hybrid assemblies and genome polishing. To evaluate small genome assemblies, we used *Escherichia coli* E2348/69, a pathovar causing diarrheal illness with a complete genome sequence including numerous plasmids (Iguchi *et al.* 2009), making it an ideal reference for testing the completeness and accuracy of bacterial and plasmid assemblies. To compare assemblies of a larger genome, we produced new long read datasets for *Drosophila ananassae* Hawaii which was previously sequenced and assembled into highly fragmented genomes (Drosophila 12 Genomes Consortium 2007; Miller *et al.* 2018). We also estimated genome size and percent heterochromatin in *D. ananassae* for comparisons with sequence assembly size. Overall, we demonstrate that no method was superior in all analyses performed, and the decision to use PacBio and ONT platforms for sequencing may depend on the specific question being addressed.

## Materials and methods

### Biological samples

*Escherichia coli* E2348/69 cultures grown overnight in LB were pelleted (12,000*g*), resuspended in 50 mM Tris, 1 mM EDTA, 10 µl RNAse (20 mg/ml) and lysed with 0.4% SDS (final) at 56°C for 30 min. A 0.5 volume of 7.5 M ammonium acetate was added, and samples were incubated for 15 min on ice. Genomic DNA was extracted with phenol: chloroform: isoamyl alcohol followed by chloroform: isoamyl alcohol and precipitated with isopropanol. After two washes with 70% ethanol, the pellet was allowed to air dry and resuspended in water.

*Drosophila ananassae* Hawaii (14024–0371.13) were originally obtained from the *Drosophila* Species Stock Center (University of California, San Diego, USA). Populations were grown on Jazz-Mix *Drosophila* food (Applied Scientific) in plastic bottles at 25°C and 70% humidity with a 12 h–12 h light–dark cycle (Klasson *et al.* 2014). Flies were treated with tetracycline to remove the *Wolbachia* endosymbiont >10 years ago (Klasson *et al.* 2014) with periodic reconfirmation of endosymbiont absence by microscopy. Genomic DNA was extracted from an unknown sex ratio of ∼350 flies with phenol: chloroform: isoamyl alcohol followed by chloroform: isoamyl alcohol and precipitated with isopropanol. After two washes with 70% ethanol, the pellet was allowed to air dry and resuspended in water.

Genomic DNA was quantified using the Qubit 4 fluorometer (Thermo Fisher Scientific) and the presence of >20 kbp fragments were validated using the FEMTO Pulse automated pulsed-field capillary electrophoresis instrument (Agilent Technologies). The same gDNA extract was used for all *E. coli* and *D. ananassae* libraries unless stated otherwise.

### Nanopore libraries and sequencing

ONT RAPID libraries for *E. coli* and *D. ananassae* were prepared with the Rapid Sequencing Kit SQK-RAD004 (Oxford Nanopore Technologies) using 400 ng DNA, 8.5 µl EB, 1.5 µl FRA, and omitting library-loading beads. After adding Rapid adapters, the reactions were incubated for 30 min at room temperature. One 24 h sequencing run was performed for *E. coli* and two sequencing runs were performed for *D. ananassae* using FLO-MIN106 R9.4.1 MinION flowcells (Oxford Nanopore Technologies).

To prepare ONT LIG libraries, gDNA was sheared to 20 kbp using g-TUBE (Covaris) and size-selected for fragments >10 kbp using the BluePippin system (Sage Science). Libraries were prepared with 1.5–3 μg size-selected DNA using the Ligation Sequencing Kit SQK-LSK109 (Oxford Nanopore Technologies) according to the manufacturer’s protocol and including 1 µl DNA control sequence (DCS) in the master mix to validate library prep. Single 24 h sequencing runs for *E. coli* and *D. ananassae* were performed with R9.4.1 MinION flowcells. An additional ONT LIG sequencing run was performed on the second *E. coli* gDNA extract using a FLO-MIN111 R10 MinION flowcell (Oxford Nanopore Technologies) that was produced without library size selection or shearing.

Base calling for all R9 runs was performed with Guppy v.4.2.2. using the “dna_r9.4.1_450bps_hac” model. Base calling for the R10 run was performed using the “dna_r10_450bps_hac” model. The default Guppy quality score cutoff >7 was used to retain “ONT pass” reads which were used for all subsequent analyses. DCS sequences were removed from ONT LIG fastq files using NanoLyse (De Coster *et al.* 2018).

### PacBio libraries and sequencing

PacBio libraries were prepared using the SMRTbell Template Prep Kit 1.0/SMRTbell Express Template Prep Kit 2.0 (Pacific Biosciences). To prepare the RS II library, genomic DNA was sheared to 20 kbp using g-TUBE as performed for ONT libraries (Covaris) followed by DNA-damage repair and end-repair using polishing enzymes. Blunt-end ligation was used to create the SMRTbell template. To prepare the Sequel II CCS/HiFi library, genomic DNA was sheared to 15 kbp using the Megaruptor 2 (Diagenode). Unsheared Sequel II CLR and sheared CCS/HiFi libraries were ligated with overhang adapters. Library fragments were size-selected using BluePippin. SMRTbell Polymerase Complex was created using DNA/Polymerase Binding Kit P6 v2 for RSII libraries, Sequel II Binding Kit 1.0 for Sequel II CLR libraries, and Sequel II Binding Kit 2.0 for Sequel II CCS/HiFi libraries (Pacific Biosciences). PacBio RS II libraries were sequenced using DNA Sequencing Reagent Kit 4.0 v2 and RS II SMRT Cells v3 (Pacific Biosciences), with 4 h movie length. Sequel II CLR libraries were sequenced using Sequel II Sequencing Plate 1.0 and SMRT Cells 8 M (Pacific Biosciences) with 15 h movie length. Sequel II CCS/HiFi libraries were sequenced using Sequel II Sequencing Plate 2.0 and SMRT Cells 8 M with 30 h movie length.

### Illumina libraries and sequencing

Illumina libraries for *E. coli* and *D. ananassae* were prepared using the KAPA HyperPrep kit (Kapa Biosystems) using manufacturer’s instructions. Quantification of libraries was performed using the Quant-iT PicoGreen dsDNA kit (Thermo Fisher Scientific). Library fragment size was assessed with the LabChip GX instrument (PerkinElmer). Paired end libraries (2x150 bp) were sequenced on an Illumina HiSeq4000 instrument (Illumina). Four additional Illumina libraries were prepared from gDNA extracted from individual flies (two male, two female) using the same methods. Adapters and low quality ends of Illumina reads were trimmed by the Institute for Genome Sciences Genomics Resource Center and inspected in FASTQC v.0.11.9 (https://www.bioinformatics.babraham.ac.uk/projects/fastqc/; last accessed February 2021).

### *Escherichia coli* genome assembly

Read length summary statistics were generated using seqkit v.0.7.2 (Shen *et al.* 2016). Read length histograms were generated from bbtools readlength.sh v.38.47 (Bushnell 2014) using 1 kbp bins. Reads from four libraries (ONT LIG, PacBio RS II, PacBio Sequel II CLR, PacBio Sequel II HiFi) were randomly downsampled to 100X sequencing depth using seqkit.

Canu assemblies were generated using v.2.1.1 (Koren *et al.* 2017) with corOutCoverage = 1000 to include all downsampled reads and genomeSize = 4.6m. The -pacbio-hifi parameter was used to invoke HiCanu (Nurk *et al.* 2020) assembly of PacBio HiFi reads. After assembly, circularization of the *E. coli* genome and candidate plasmids was attempted by the “minimus2” command of Circlator v.1.5.5 (Hunt *et al.* 2015) followed by *E. coli* genome rotation using the Circlator “fixstart” command. Genomes were polished for one round using Pilon v.1.22 (Walker *et al.* 2014) with 100X Illumina reads and the parameters --minmq 10 and --fix bases.

Flye assemblies were generated using v.2.8.2 (Kolmogorov *et al.* 2019) with the --plasmids parameter to attempt to assemble plasmids and -i 1 set as default to polish assemblies for a single round with long reads. The--pacbio-hifi parameter was used to assemble PacBio HiFi reads.

Hybrid Unicycler assemblies were generated using v.0.4.8 (Wick *et al.* 2017b) with long reads and 100X Illumina data. The Illumina dataset was generated by first removing sequencing duplicates using bbtools clumpify.sh followed by random downsampling with seqkit. The Unicycler pipeline includes a polishing step, performed here using Pilon.

### Evaluation of *E. coli* genome assemblies

Assembly characteristics were evaluated using QUAST v.5.0.2 (Mikheenko *et al.* 2018) with the published *E. coli* E2348/69 genome assembly (GenBank GCA_000026545.1) set as the reference sequence. The presence of circularized sequences was assessed in the corresponding assembly output reports. To validate an ∼18 kbp deletion event in the assemblies generated in this study relative to the published genome sequence, long reads were mapped to GCA_000026545.1 using minimap v.2.17 (Li 2018) and Illumina reads were mapped using bwa-mem v.0.7.17 with -k 23 (Li 2013) and visualized in IGV v.2.3.81 (Thorvaldsdóttir *et al.* 2013). In addition, primers were designed for two genes from GCA_000026545.1 in deletions and two flanking region genes. PCR reactions (25 μl) consisting of 0.2 μM forward primer, 0.2 μM reverse primer, 10 ng of DNA, and 1X Taq 2X Master Mix (NEB) were initially denatured at 95°C for 30 s. The samples underwent 30 cycles consisting of a 30 s 95°C denaturation phase, 30 s 56.5°C annealing phase, and a 60 s 68°C extension phase. The PCR products were extended with a final 5 min 68°C extension phase. After amplification, PCR amplicons were visualized on a 1.5% agarose gel run at 125 V for 1 h with 100 bp Bioline Hyperladder. PCR products were Sanger sequenced with forward and reverse primers by Genewiz. The presence of highly conserved genes was determined using BUSCO v.4.0.6 (Simão *et al.* 2015; Waterhouse *et al.* 2018) using the bacteria odb10 dataset from OrthoDB (Kriventseva *et al.* 2019). To estimate consensus identity values for each assembly, the longest genome contig was extracted and aligned to a concatenated 2X copy of a consensus *E. coli* genome sequence produced by three separate Unicycler assemblies (referred to as Ecoli.UMIGS) using QUAST. The number of mismatches and indels per 100 kbp were reported by QUAST, and consensus identity was estimated by the formula (100,000-(mismatch rate + indel rate))/100,000.

To evaluate chimeric read content in sequencing datasets, long reads were aligned to Ecoli.UMIGS using minimap2. Output files in paf format were used to identify putative chimeras using Alvis (Martin and Leggett 2019). Using the -chimeras parameter, a read was called as chimeric when ≥90% of its length overlapped the consensus genome (-minChimeraCoveragePC 90) and two sub-alignments ≥10% of the total read length aligned to discordant regions of the genome (-minChimeraAlignmentPC 10). Since reads mapping to the two ends of the linear representation of the *E. coli* genome would be identified as chimeric, a second run of Alvis was performed with a rotated genome. Putative chimeras were calculated as the number of reads assigned as chimeras in both Alvis runs. Alignments were manually inspected in IGV (Thorvaldsdóttir *et al.* 2013).

### *Escherichia coli* plasmid composition analysis

To detect plasmids in sequencing datasets, we submitted polished assemblies to the plasmid database PLSDB (Galata *et al.* 2019) using the mash dist search strategy with default parameters. To assess sequencing depth and estimated plasmid copy number, long reads were mapped to the consensus Unicycler genome using minimap2 (Li 2018) with default parameters and Illumina reads were mapped using bwa-mem with -k 23 (Li 2013). Picard v.2.5.0 (https://broadinstitute.github.io/picard/; last accessed February 2021) was used to remove duplicate Illumina alignments, and SAMtools v.1.9 (Li *et al.* 2009) was used to calculate sequencing depth for each position in the reference genome. The estimated copy number for the *E. coli* genome and plasmids was determined by dividing the total number of bases mapping to each sequence by the total length of the sequence. As a separate test of plasmid copy number, primers were designed for the *E. coli* genome, pMAR2, and p5217 from the Unicycler consensus assembly and pE2348-2 from NCBI (GenBank FM18070.1). Amplicons were quantified using the CFX384 Touch Real-Time Detection System and qPCR cycle threshold and melt curve values were obtained from CFX Maestro Software (Bio-Rad Laboratories Inc.). The mean cycle threshold (Ct) value for each sequence was calculated by averaging values from three replicates. ΔCt was calculated as the difference between the mean Ct value of the sequence of interest and the mean Ct genome. Estimated sequence copy number was calculated as 2^-ΔCt^ (Livak and Schmittgen 2001). As a negative control, qPCR experiments also included samples with no template DNA.

### *Drosophila ananassae* genome size and percent heterochromatin estimations

The genome size was estimated for each sex of the *Wolbachia*-cured *D. ananassae* Hawaii (14024–0371.13) strain using flow cytometric methods (Johnston *et al.* 2019). In brief, neural tissue from individuals of each sex (female *n* = 5, male *n* = 4) was dissected and co-prepared with neural tissue from female *D. virilis* standard (Johnston lab strain, 328 Mbp) and placed into 1 mL of Galbraith buffer. Samples and standards were ground with a “loose” Kontes “A” pestle 15 times in order to release nuclei. Samples were then passed through a 40-μm mesh filter before staining in cold and dark for at least 120 min with 25 μL of 1 mg/μL propidium iodide. Mean fluorescence was determined for the 2C (diploid) peaks produced by nuclei of the sample and the standard using a Cytoflex flow cytometer (Beckman Coulter). Genome size was estimated as a ratio of the mean fluorescence of sample versus standard, multiplied by the amount of DNA in the standard. The proportion of thoracic underreplication was estimated as previously described (Johnston *et al.* 2013; Hjelmen *et al.* 2020; Johnston *et al.* 2020). Briefly, thoraces of both male and females were individually dissected, ground, filtered, stained, and scored with a Cytoflex flow cytometer as described above. The mean channel number of peaks for 2C (diploid) and underreplicated nuclei were calculated for each individual. Percent underreplication values were calculated by subtracting the 2C value from the underreplication value, then dividing by the 2C value.

### *Drosophila ananassae* genome assembly

Read length summaries and histograms were generated using seqkit and bbtools as discussed above. The ONT and PacBio Sequel II CLR libraries were assembled with Canu v.2.1.1 using genomeSize = 240m (240 Mbp) and default assembly parameters. All ONT datasets were combined prior to assembly. The PacBio Sequel II HiFi reads were assembled with -pacbio-hifi to invoke HiCanu. The genomeSize parameter determines the selection of the longest input reads up to ∼40X sequencing depth and was chosen for *D. ananassae* to account for the inclusion of one set of autosomes, chromosome X, and chromosome Y. The value is larger than the 1C flow cytometric estimates for females, which is the size of one set of autosomes and one X; the 1C estimate for males is the size of one set of autosomes and the average size of chromosomes X and Y. A hybrid assembly was also generated with combined read data from ONT and CLR reads. Libraries were assembled with Flye using the parameters -g 240 m and --asm-coverage 40. ONT and CLR reads were assembled in raw read mode, while HiFi reads were assembled in hifi mode. Assemblies were polished for a single round as the default Flye parameter. To test the impact of short read polishing, ONT Canu and ONT Flye assemblies were polished with Pilon v.1.22 for one round with --minmq 10 and --fix bases.

### Dana.UMIGS genome assembly

A new *D. ananassae* reference assembly (referred to as Dana.UMIGS) was generated to enable comparisons between test assemblies produced in this study. The six longest chromosome arm contigs were extracted from various assemblies (XL, XR, 3L from Canu PacBio CLR; 2L, 2R from Flye PacBio CLR; 3R from Flye ONT). After removal of chromosome arm contigs from the Canu PacBio CLR assembly, the remaining contigs were combined with the six contigs described above. Following the merger of the Canu CLR (recipient) and HiCanu HiFi contigs >50 kbp (donor) assemblies with quickmerge v.0.3 (Chakraborty *et al.* 2016) using conservative parameters (-ml 5,000,000 -l 20,000), the resulting merging events were manually inspected. The output assembly was polished for one round with Arrow v.2.3.3 (SMRTTools v7) using CLR reads and Pilon for two rounds using HiFi reads with the parameters --minmq 10 and --fix bases. To assess the presence of duplicated content, HiFi reads were mapped to the assembly using minimap2 and a histogram of sequencing depth across the genome was produced using purge_haplotigs v.1.1.1 (Roach *et al.* 2018). After manually inspecting assembly contigs classified by purge_haplotigs, we removed 79 contigs corresponding to bacterial contaminants, assembly artefacts, and erroneous duplications in the assembly.

### Anchoring Dana.UMIGS contigs

To identify contigs in the Dana.UMIGS assembly corresponding to the major euchromatic chromosomes (X, 2, 3), regions with known positions on chromosome arms were extracted from the *D. ananassae* caf1 assembly (Drosophila 12 Genomes Consortium 2007). Coordinates of the caf1 regions were reported previously (Schaeffer *et al.* 2008). Dana.UMIGS assembly contigs were searched for caf1 sequences using BLASTN v.2.10.0 (Altschul *et al*. 1990; Camacho *et al*. 2009). After initial searches using default parameters indicated the presence of high-quality matches as the first hits for each caf1 query, a second BLASTN search was conducted to retain the single best hit for each query (-max_target_seqs 1 -max_hsps 1). The positions of loci in the Dana.UMIGS assembly were plotted using a custom script in R and connected to *D. ananassae* polytene maps from (Tobari 1993).

To anchor contigs to chromosome Y, reads from two male and two female Illumina libraries were randomly downsampled to ∼150X depth using seqkit and mapped to the Dana.UMIGS assembly using bwa mem. Duplicate reads were removed with Picard and BAM files were subsequently merged to generate single files for males and females. The sequencing depth for each position in the genome was determined with SAMtools depth while removing low-quality mappings (-Q 10). A script adapted from Chang & Larracuente (2019) was used to split the genome into 10 kbp windows and determine the median female/male sequencing depth ratio for each window. Contigs were assigned as putative Y contigs as having at least one window with a median female/male ratio of zero and ≥80% of its windows with median female/male ratios below 0.05.

To anchor contigs to chromosome 4, Dana.UMIGS contigs were aligned to caf1 assembly contigs previously assigned to chromosome 4 (Leung *et al.* 2017) using NUCmer (Kurtz *et al.* 2004) with -l 1000. Chromosome 4 contigs containing LGT from the fly’s *Wolbachia* endosymbiont (*w*Ana) were anchored by aligning Dana.UMIGS contigs to the previously assembled *w*Ana genome (Gasser *et al.* 2019) with -l 1000. Alignments were filtered to retrieve the longest consistent set of alignments for each LGT contig. To estimate for the amount of LGT present in each assembly, the number of positions in LGT contigs aligning to the *w*Ana genome was determined using BEDtools v.2.27.1 (Quinlan and Hall 2010).

### Evaluation of *D. ananassae* genome assemblies

To generate assembly statistics, assembly contigs were evaluated using QUAST with the --large parameter (Mikheenko *et al.* 2018). K-mer completeness values of each assembly and assembly spectra copy number plots were generated using KAT v.2.4.0 (Mapleson *et al.* 2016). BUSCO searches were conducted using the arthropoda odb10 dataset (Kriventseva *et al.* 2019). For comparisons to published *D. ananassae* assemblies, the same analyses were performed on the caf1 genome assembly (GenBank GCA_000005115.1) (Drosophila 12 Genomes Consortium 2007) and a genome assembled by Miller *et al.* (2018) using ONT sequencing.

To evaluate contiguity of the euchromatic chromosome arms in assemblies produced from single long read libraries, contigs corresponding to chromosome X, 2, and 3 were extracted from each assembly using minimap2-based alignments >50 kbp. Dana.UMIGS contigs were aligned to contigs sets with nucmer using -l 500 and --maxmatch. Contiguity and consensus identity metrics of chromosome arm contigs were determined with QUAST by setting the sequences from Dana.UMIGS assembly as the reference.

To assess library composition bias in sequencing euchromatic and heterochromatic reads, ONT and PacBio reads were mapped to the Dana.UMIGS assembly using minimap2, and Illumina libraries were mapped using bwa mem. After removal of secondary and supplementary alignments with SAMtools, the sequencing depth of libraries across each contig was determined using the purge_haplotigs “hist” command. Distributions of sequencing depth for each library were plotted for the entire genome and contigs anchored to chromosome Y, chromosome 4, and *w*Ana LGT contigs. To obtain depth distributions for euchromatic and heterochromatic regions, BAM files were subsetted with SAMtools to retain reads mapping on chromosome X, 2, and 3 coordinates; euchromatic regions were based on coordinates of genes from the *D. ananassae* polytene map and heterochromatic regions were approximated as all remaining contig intervals. The depth of euchromatic and heterochromatic regions was determined by running purge_haplotigs “hist” on subsetted BAM files.

### DNA modification

Detection of DNA methylation using *E. coli* PacBio libraries was assessed with PacBio SMRT Tools. Differences in RS II and Sequel II CLR libraries necessitated the use of similar pipelines (*i.e.*, PacBio base modification pipeline) on different software releases (RS II: SMRT Link v.7; Sequel II CLR: SMRT Link v.8). Reads were mapped to the *E. coli* genome with pbmm2 and detection of DNA methylation signatures was performed with ipdSummary using –identify m4C, m6A, m5C_TET to search for m4C, m6A, and m5C modifications, respectively. Highly modified motifs were identified with motifMaker. The distribution of modification QV scores for the four nucleotide bases was produced by the SMRT Tools pipeline and an appropriate modification QV cutoff was determined.

Detection of DNA methylation using *E. coli* ONT libraries was assessed with Tombo v.1.5 (Stoiber et al. 2016) using the *de novo* model for modified base detection. The dampened fraction of reads supporting each modification event was produced with Tombo text_output. The presence of DNA modification at specific motifs was assessed using the Tombo plot motif_with_stats command by plotting the dampened fraction (raw fraction plus pseudo-counts to un-modified and modified read counts) values for up to 10,000 genomic sites containing the motif of interest. ROC curves for detected GATC and CCWGG motifs in ONT libraries were produced with the tombo plot roc command.

To assess the consistency of m6A modification calls using PacBio and ONT reads, the modified base detection of ONT reads was performed using the m6A alternate model followed by the retrieval of the dampened fraction of read support using Tombo. The wig2bed command of BEDOPS v.2.4.36 (Neph *et al.* 2012) was used to convert Tombo output. The resulting BED file containing dampened fraction values was cross-referenced against the motifs.gff output file of SMRT Tools to retrieve PacBio Sequel II CLR modification QV scores at the same *E. coli* genome coordinates. Of 48,664 total m6A modifications assessed by both pipelines, a random subsample of 5000 was plotted to visualize whether modifications were supported by both technologies.

DNA methylation detection in the *D. ananassae* ONT LIG library was conducted using Tombo similar to the methods described in *E. coli*. Given the lack of known methylated motifs in *D. ananassae*, *de novo* modified base detection was followed by the extraction of 1000 regions in Dana.UMIGS showing the largest estimated dampened fraction of modified bases using the tombo text_output command. The presence of overrepresented motifs in candidate modified regions was evaluated with MEME v.4.12.0 (Bailey *et al.* 2015) using the parameters -dna -mod zoops -nmotifs 50.

### Data availability

Raw reads supporting the conclusions of this article have been deposited in GenBank/EMBL/DDBJ Sequence Read Archive under BioProject PRJNA602597. Supplementary data files are available at figshare: https://doi.org/10.25387/g3.14096897. File S1 provides detailed descriptions of all supplementary data files. File S2 contains Tables S1–S11. File S3–S23 contains *E. coli* genome assemblies. File S24–S33 contains *D. ananassae* genome assemblies. File S34–S36 contain code for data processing and visualization. All commands and scripts used in the study are available at https://github.com/Dunning-Hotopp-Lab/Ecoli-Dana-LongReads.

## Results

### Read composition and *de novo* assembly performance in *E. coli*

**Figure 1:**
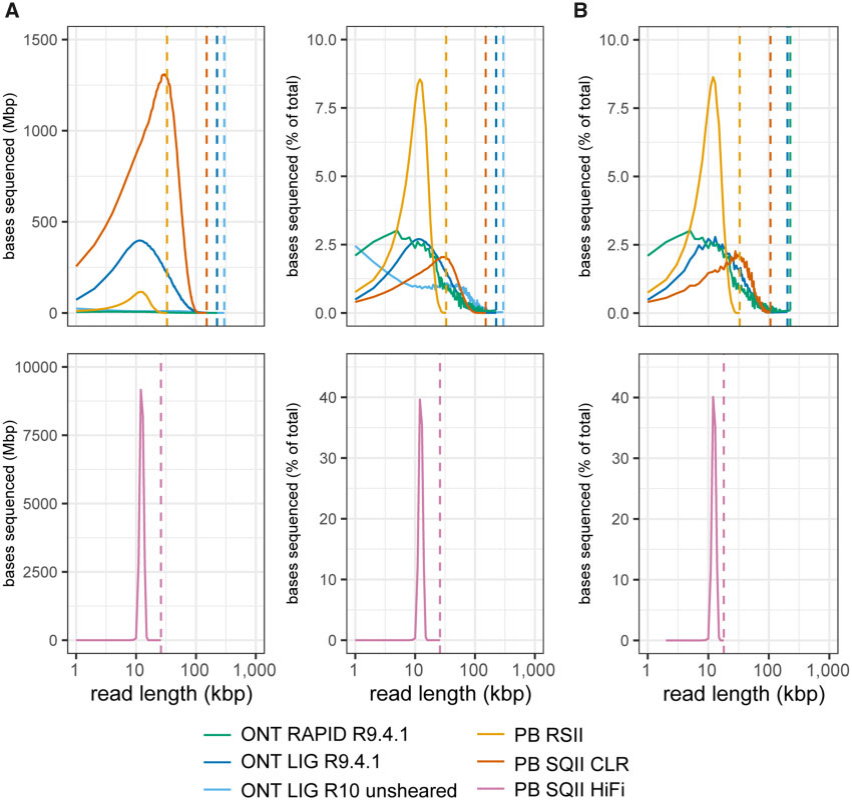
Read composition of *E. coli* long read libraries. Bases sequenced per read length were calculated for 1 kbp bins in each library. Sequenced bases are shown as (A) raw numbers and percentages for complete datasets and (B) percentages for random subsamples of 100X sequencing depth. Vertical dotted lines correspond to maximum read length for each library.

*E. coli* long-read sequencing data was generated from multiple PacBio and ONT methods (Table 1). The PacBio Sequel II CLR library had the highest total throughput and read N50 value, representing a substantial improvement compared to the older PacBio RS II. The relatively uniform distributions of the PacBio HiFi library (multipass sequencing of 10 kbp size-selected fragments) produced >17 times the amount of sequencing data relative to RS II but approximately one third of the throughput of the CLR run (Table 1; Figure 1A). Reads passing the default Guppy basecalling quality filters in the two ONT libraries had similar read N50 values (20–22 kbp) but 50-fold less total sequencing data was obtained from the RAPID run. The maximum read length for ONT libraries were about 50 kbp longer than PacBio CLR, representing a ∼33% increase in these runs (Table 1).

**Table 1:** Summary of long-read sequencing datasets used in this study

Organism	Library	Reads	Bases (Mbp)	Sequencing depth	N50 (bp)	Longest (bp)	SRA accession
*E. coli*	ONT RAPID^a^	26,079	236	47X	20,553	226,030	SRR11523179
*E. coli*	ONT LIG^a^	983,320	14,711	2,942X	23,863	225,855	SRR13679197
*E. coli*	ONT LIG^a^^c^	96,277	995	199X	46,613	297,344	SRR12801740
*E. coli*	PacBio RS II	144,238	1359	272X	12,409	33,017	SRR11434956
*E. coli*	Pacbio Sequel II CLR	3,383,930	63,970	12,794X	33,061	151,906	SRR11434960
*E. coli*	Pacbio Sequel II HiFi^b^	1,789,131	23,122	4624X	12,949	26,294	SRR11434954
*D. ananassae*	ONT RAPID^a^^d^	1,369,030	7986	33X	11,648	194,086	SRR11486455 SRR13679254 SRR13610617
*D. ananassae*	ONT LIG^a^	508,728	8126	34X	25,857	270,071	SRR13679196
*D. ananassae*	PacBio RS II	105,438	896	4X	11,910	30,524	SRR11442120
*D. ananassae*	Pacbio Sequel II CLR	4,014,577	79,786	332X	35,023	144,753	SRR11442116
*D. ananassae*	Pacbio Sequel II HiFi^b^	2,391,195	24,601	103X	10,329	21,776	SRR11442117

a Statistics are reported for “ONT pass” reads with a minimum q-value of 7.

b Statistics are reported for circular consensus sequences (CCS) of the PacBio HiFi sequencing runs.

c To validate plasmid composition results, one additional *E. coli* ONT LIG sequencing run was performed using the R10 pore without library size selection or shearing.

d Statistics are reported for combined *D. ananassae* ONT RAPID runs using the same sequencing flowcell version and library preparation methods.

Because we did not make an effort to sequence at the same read depth in all libraries, the distributions of sequenced bases largely reflect differences in the total sequencing throughput of each run (Figure 1A). To enable *de novo* assemblies using similar amounts of input sequencing data, reads were randomly downsampled to 100X depth while maintaining read length distributions observed in the full datasets (Figure 1B). An exception was the ONT RAPID library which was assembled with the full 47X sequencing depth produced in the run. *E. coli* assemblies were produced from these random subsets (1) using Canu (Koren *et al.* 2017; Nurk *et al.* 2020) with long reads alone, (2) using Flye (Kolmogorov *et al.* 2019) with long reads, and (3) using Unicycler (Wick *et al.* 2017b) with a hybrid approach combining the long reads with 100X Illumina reads.

Of the 15 *E. coli* assemblies produced in this study, nine contained a single *E. coli* genome contig that was assigned as circular by the assembler (Table 2; Supplementary Table S2). The bacterial genome size converged to ∼5 Mbp when using different libraries and assemblers, similar to the size of the published genome sequence for this strain (Iguchi *et al.* 2009). While the ONT RAPID and PacBio CLR datasets were assembled into circularized genomes by all three assemblers, the ONT LIG, PacBio RS II, and PacBio HiFi datasets each were assembled into a circularized genome only once. When assembly contigs were aligned to the genome sequence for this *E. coli* strain (GenBank FM180568.1) using QUAST (Mikheenko *et al.* 2018), there was evidence of assembly fragmentation specific to individual read libraries as well as two inversion regions that were problematic for multiple assemblers and read libraries. These regions contain phage tail assembly proteins that are known to have polymorphic inversions within *E. coli* populations (Forde *et al.* 2014), and there is read support for both orientations in one of these regions (Supplementary Figure S1). The consensus *E. coli* genome sequenced in this study was also ∼21 kbp shorter than the published genome sequence for this strain (Table 1). To validate the genome reduction, we focused on an ∼18 kbp region that was present in the NCBI sequence but absent in the Unicycler assembly (Supplementary Figure S2A). The junction spanning the deletion region was validated with PCR amplification (Supplementary Figure S2B), and genes apparently absent from this *E. coli* specimen are involved in colanic acid biosynthesis (Supplementary Table S3).

**Table 2:** Summary of *E. coli* E2348/69 assemblies

Library 1	Assembler	Total contigs	Largest genome contig	Largest pMAR2 contig	Largest p5217 contig	BUSCO^b^ (%)	Consensus identity^c^ (%)
ONT RAPID	Canu	6	**4,989,389**	**189,389**	**11,738**	91.13	99.950
ONT RAPID	Canu^a^	4	**4,944,380**	**96,603**	**10,423**	100.00	99.997
ONT RAPID	Flye	3	**4,943,164**	**96,555**	**5212**	93.55	99.972
**ONT RAPID**	**Unicycler**	7	**4,944,462**	**96,603**	**5218**	100.00	NA
ONT LIG	Canu	4	3,093,902	**141,938**	NA	92.74	99.967
ONT LIG	Canu^a^	4	3,094,900	**96,602**	NA	100.00	99.996
ONT LIG	Flye	2	3,402,910	NA	NA	93.55	99.974
**ONT LIG**	**Unicycler**	7	**4,944,462**	**96,603**	**5218**	100.00	NA
PB RS II	Canu	72	265,067	28,923	NA	45.97	99.747
PB RS II	Canu^a^	67	265,619	29,066	NA	93.55	99.979
PB RS II	Flye	5	**4,941,598**	**96,381**	NA	79.84	99.898
PB RS II	Unicycler	13	4,885,846	**95,943**	**5218**	100.00	NA
PB SQ II CLR	Canu	4	**4,989,961**	**132,660**	NA	99.19	99.998
PB SQ II CLR	Canu^a^	3	**5,044,086**	**96,604**	NA	100.00	99.997
PB SQ II CLR	Flye	2	**4,944,307**	**96,604**	NA	100.00	99.997
**PB SQ II CLR**	**Unicycler**	7	**4,944,462**	**96,603**	**5218**	100.00	NA
PB SQ II HiFi	HiCanu	56	4,930,997	**109,122**	NA	100.00	99.999
PB SQ II HiFi	HiCanu^a^	10	4,931,051	**96,603**	NA	100.00	99.998
PB SQ II HiFi	Flye HiFi	2	**4,944,462**	**96,603**	NA	100.00	99.999
PB SQ II HiFi	Unicycler	13	4,885,847	**96,603**	**5218**	100.00	NA
BAC clones (Iguchi *et al.* 2009)	Phrap (de la Bastide and McCombie 2007)	3	4,965,553	97,978	NA	100.00	99.996

Bolded library/assembler combinations produced an identical set of genome and plasmid sequences and were treated as ground truth for other analyses (Ecoli.UMIGS). Bolded numbers indicate sequences putatively assigned as circular by the assembler.

a Results reported from polished and circularized Canu assemblies.

b The sum of complete and duplicated BUSCOs recovered using the bacteria odb10 dataset (124 total).

c Mismatch and indel rates per 100 kbp were calculated by comparing the longest genome contig in Canu/Flye assemblies to the Ecoli.UMIGS assembly in QUAST.

NA, not applicable.

Three Unicycler hybrid assemblies using ONT RAPID, ONT LIG, and PacBio CLR reads produced an identical circular genome sequence (referred to as Ecoli.UMIGS) and was treated as ground truth for comparisons of other assemblies in this study. Contaminants were present in these Unicycler assemblies that were removed following assembly; the Illumina libraries contain contaminating reads, as is common, and in this case included reads from human, *Neisseria gonorrhoeae*, and an unknown bacteria.

### *E. coli* assembly quality

One method to characterize *E. coli* assembly quality is the presence of highly conserved bacterial genes, assessed here using BUSCO (Simão *et al.* 2015; Waterhouse *et al.* 2018). The complete set of 124 bacterial BUSCO genes was successfully characterized in eight assemblies (Table 2; Supplementary Table S2). When evaluating Canu and Flye assemblies produced by long reads alone, BUSCO characterization was superior in PacBio Sequel II assemblies (CLR and HiFi) and the worst in RS II assemblies, with ONT libraries producing results more similar to Sequel II. Utilization of the Illumina reads to polish the Canu assemblies or construct hybrid Unicycler assemblies improved the characterization of complete BUSCOs (Table 2; Supplementary Table S2).

Consensus identity values of Canu and Flye assemblies were estimated by aligning the longest assembled genome contig against a 2X concatenated copy of the Ecoli.UMIGS genome sequence using QUAST. Unicycler assemblies were excluded from this analysis due to the circular reasoning of the comparisons involved. All assembled genomes had >99% consensus identity to Ecoli.UMIGS, and when excluding PacBio RS II assemblies the remaining had >99.9% consensus identity. Mismatches per 100 kbp of aligned sequence were similar across read libraries and assemblies, whereas indels were more prevalent in ONT and PacBio RS II assemblies. Systematic, non-random errors in ONT sequencing data may cause lower consensus accuracy (Giordano *et al.* 2017). Polishing ONT and RS II assemblies with Illumina data reduced the incidence of indel errors. PacBio Sequel II CLR and HiFi contigs produced sequences with >99.99% consensus identity values, with HiFi displaying slightly better performance with the use of HiFi parameters in Canu/Flye. Mismatch errors increased in the Canu CLR assembly and indel errors increased in the Canu CLR/HiFi assemblies following the circularization and Illumina polishing pipeline, possibly owing to (1) issues arising from automated circularization strategies, and/or (2) improper resolution of repetitive genome regions using short read polishing.

The resolution of the *E. coli* genome into a single contiguous sequence enabled the assessment of the presence of chimeric reads in long read libraries. After mapping reads to the Ecoli.UMIGS assembly, chimeras were quantified using Alvis (Martin and Leggett 2019). The ONT RAPID library had the lowest percentage of putative chimeric reads (0.02%), while ONT LIG had the highest (3.17%), (Supplementary Table S3). Visualization of alignments in IGV (Thorvaldsdóttir *et al.* 2013) did not suggest an artificial inflation of chimeras (Supplementary Figure S3).

### *Escherichia coli* plasmids are underrepresented in ligation-based long read libraries

The presence of the ∼97 kbp plasmid pMAR2 and the ∼5 kbp plasmid p5217 was confirmed with searches against the plasmid database PLSDB (Galata *et al.* 2019). Both plasmids were recovered when assembling with ONT RAPID or Illumina reads (Table 2). The pMAR2 plasmid contig was assembled up to 2X-longer than the actual plasmid with Canu, but this could be resolved with polishing and circularization (Table 2). The p5217 plasmid was not assembled with only PacBio reads, the pMAR2 was not assembled with ONT LIG reads using Flye, and the previously reported pE2348-2 plasmid from this *E. coli* strain (GenBank FM18070.1) (Iguchi *et al.* 2009) was not present in any assemblies.

Size-selected libraries may fail to produce plasmid sequences in assemblies if plasmid sizes are small, but assemblers could also fail to properly identify plasmids because of k-mer abundance differences due to copy-number differences. To differentiate between these scenarios, reads were mapped to the Ecoli.UMIGS reference assembly. The combined depth of pMAR2 and p5217 contributed to 5% of total sequencing depth in ONT RAPID libraries with both being estimated to be present in 2-3X copy number relative to the *E. coli* genome (Supplementary Table S5). Sequence reads from plasmids were less abundant in other libraries, contributing to less than 2% of total sequencing depth in ONT LIG and PacBio datasets. The size-selected PacBio libraries had no primary alignments to the p5217 plasmid.

Using qPCR, there are an estimated two copies of p5217 per genome, similar to the observed ratio in the ONT RAPID reads (Supplementary Tables S6 and S7; Supplementary Figure S4) while there is an estimated single copy of pMAR2 per genome. Assuming that the qPCR results are correct, this suggests that pMAR2 is overrepresented in the ONT RAPID library and underrepresented in ONT LIG and PacBio libraries, although amplification biases have been demonstrated in plasmid qPCR experiments (Lin *et al.* 2011). The results for the pE2348-2 replicates resembled the negative control, suggesting this plasmid is not present in this *E. coli* sample (Supplementary Figure S5). These findings are consistent with previous observations of underrepresentation of small plasmid sequences in size-selected libraries (George *et al.* 2017; Wick *et al.* 2017a).

The second ONT LIG run performed on a R10 flowcell produced a lower throughput of sequencing data (∼1.0 Gbp) compared to the R9.4.1 run and had a larger proportion of bases sequenced in reads <10 kbp (Table 1; Figure 1A). Mapping rates of sequences from the two ONT LIG flow cells were similar (R9.4.1: 99.87%; R10: 99.63%; Supplementary Table S5). The recovery of plasmid reads was superior in the ONT LIG R10 library but both plasmids remained underrepresented (Supplementary Table S5). Overall, all but ONT RAPID gave lower than expected plasmid sequencing depth ratios while ONT RAPID was the only protocol to consistently produce reads corresponding to small plasmids.

### Detecting DNA modification using *E. coli* long read libraries

According to REBASE (Roberts *et al.* 2015), *E. coli* E2348/69 has ten methyltransferases and three methylated motifs, including m6A modification of 5′-GATC-3′ by DNA adenine methyltransferase (Dam) and its three paralogs, m6A modification of 5′-ATGCAT-3′ by YhdJ DNA methyltransferase, and 5mC modification of 5′-CCWGG-3′ by DNA cytosine methyltransferase (Dcm) (Marinus and Løbner-Olesen 2014).

Base modifications are detected in PacBio sequencing data as an increased time interval between fluorescent pulses emitted by successive base incorporations, known as interpulse duration (IPD). Using the PacBio RS II library, the SMRT Tools DNA base modification pipeline identified five DNA motifs forming three distinct palindromes that were only enriched for m6A methylation (Supplementary Table S8). There were 39,674 GATC sites identified in the *E. coli* dsDNA genome sequence, and >99.8% were characterized as methylated (Supplementary Table S8), likely by Dam and its paralogs. Most GATC sites assigned as unmethylated were noncoding (Supplementary Table S9), in agreement with the observation of methylase protection in noncoding regions in a previous study (Tavazoie and Church 1998). The YTCAN^6^GTNG/CNACN^6^TGAR motif had 880 sites with nearly ubiquitous methylation. This DNA methylase recognition sequence is shared with four REBASE entries, including three *E. coli* strains and *Shigella boydii* ATCC 49812 but was not previously characterized in this *E. coli* strain. The CYYAN^7^RTGA/TCAYN^7^TRRG motif had 579 sites and was nearly universally methylated but had no matches in REBASE (Supplementary Table S8).

The greater sequencing depth of the Sequel II library resulted in higher modification quality values (QV) and GV was a nondescript motif reported by default SMRT Tools parameters (Supplementary Table S8). Guanine nucleotides had exceptionally high baseline modification QV, indicative of bias in the v.0.9 beta sequencing chemistry likely resulting in spurious identification of GV as a methylated motif (Supplementary Figure S5). This bias was not unexpected since base modification was not supported in this chemistry release during the beta testing phase when this sequencing was completed. However, at the suggestions of Pacific Biosciences, increasing the modification QV threshold removed the GV motif from the report but identified fewer methylated GATC sites (99.3%; Supplementary Table S8). Elevated baseline modification QV scores were not observed in RS II data (Supplementary Figure S5).

**Figure 2:**
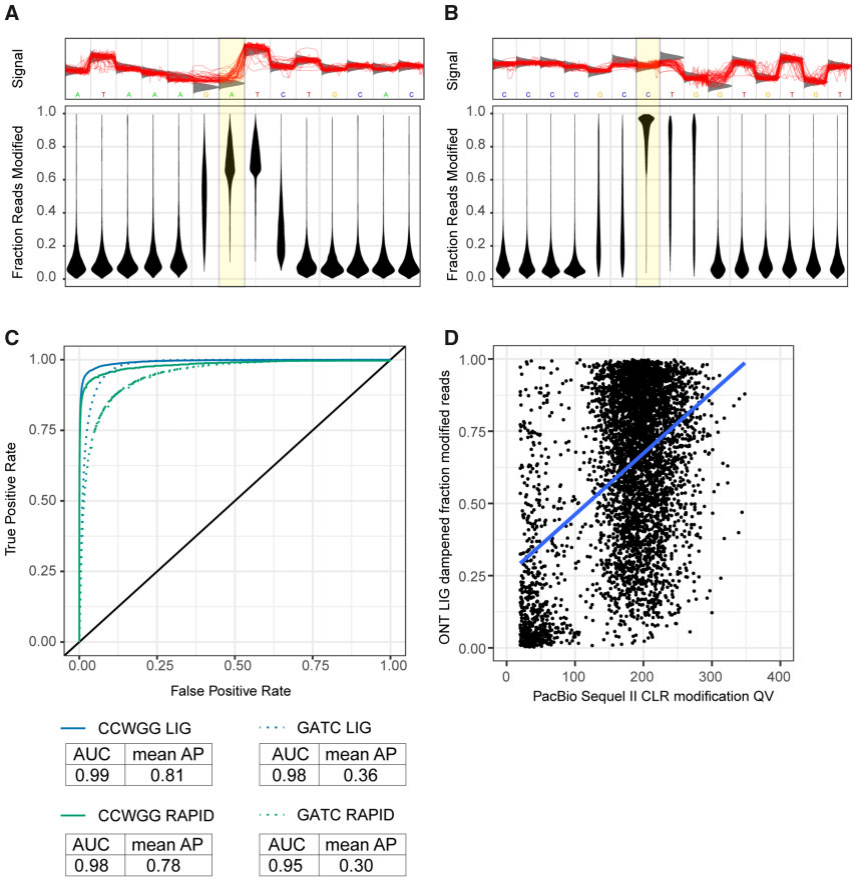
Evidence of DNA methylation in *E. coli* E2348/69 using long-read sequencing. Methylation at (A) GATC and (B) CCWGG motifs are supported using ONT LIG sequencing. Top: an example motif is shown, with individual reads plotted to the region shown in red. The expected raw signal distribution using a canonical base model (=unmethylated DNA) is shown in grey. The location of known methylation in *E. coli* is highlighted. Bottom: the fraction of reads supporting a modification event is reported for each position in the motif, and the distribution of proportions are shown. Higher values indicate the motif is more ubiquitously methylated in the *E. coli* genome. Distributions are shown for 11,313 GATC motifs and 20,063 CCWGG motifs. (C) ROC curves for detection of methylation at known motifs. GATC and CCWGG motifs were considered ground truth and modified base statistics of these sites were compared against statistics at other base modification sites. ROC curves for ONT RAPID (∼60X depth) and ONT LIG (∼3280X) are plotted with corresponding area under the curve (AUC) and average precision (AP) values for each condition shown. (D) Association of m6A modifications assessed using PacBio Sequel II CLR and ONT LIG sequencing. All m6A modifications with a PacBio modification QV >20 were cross-referenced for corresponding dampened fraction values in ONT LIG sequencing. A random sample of 5000 m6A modifications are plotted (total = 48,664). A linear regression was fitted to the data.

DNA methylation in *E. coli* ONT libraries was assessed with Tombo (Stoiber et al. 2016). Tombo uses canonical base models for *de novo* detection of DNA methylation events in addition to alternative models that are modification-centric (*e.g.*, m5C, m6A) or motif-centric (*e.g.*, GATC, CCWGG). The *de novo* model identified a high incidence of methylation activity at the GATC, YTCAN^6^GTNG, and TCAYN^7^TRRG palindromic motifs similar to the PacBio sequencing (Figure 2A; Supplementary Figure S6). There was no apparent support for methylation at ATGCAT motifs, which is consistent with the PacBio results (Supplementary Figure S6) and unsurprising given a previous characterization of YhdJ as a nonessential methyltransferase (Broadbent *et al.* 2007). Unlike PacBio, ONT sequencing identified cytosine methylation at CCWGG motifs (Figure 2B). Detection of methylated GATC and CCWGG motifs improved with the increased sequencing depth of the ONT LIG data, supporting previous findings (Figure 2C) (Oxford Nanopore Technologies 2018).

Due to the distinct pipelines of SMRT Tools and Tombo, it is difficult to assess the convergence of DNA modification using PacBio and ONT sequencing. In addition to the joint identification of specific m6A-methylated motifs, there is a positive association between PacBio Sequel II m6A modification QV and the proportion of ONT LIG reads supporting a m6A methylation event at adenine residues across the *E. coli* genome (Figure 2D).

### Read composition and *de novo* assembly performance in *D. ananassae*

**Figure 3:**
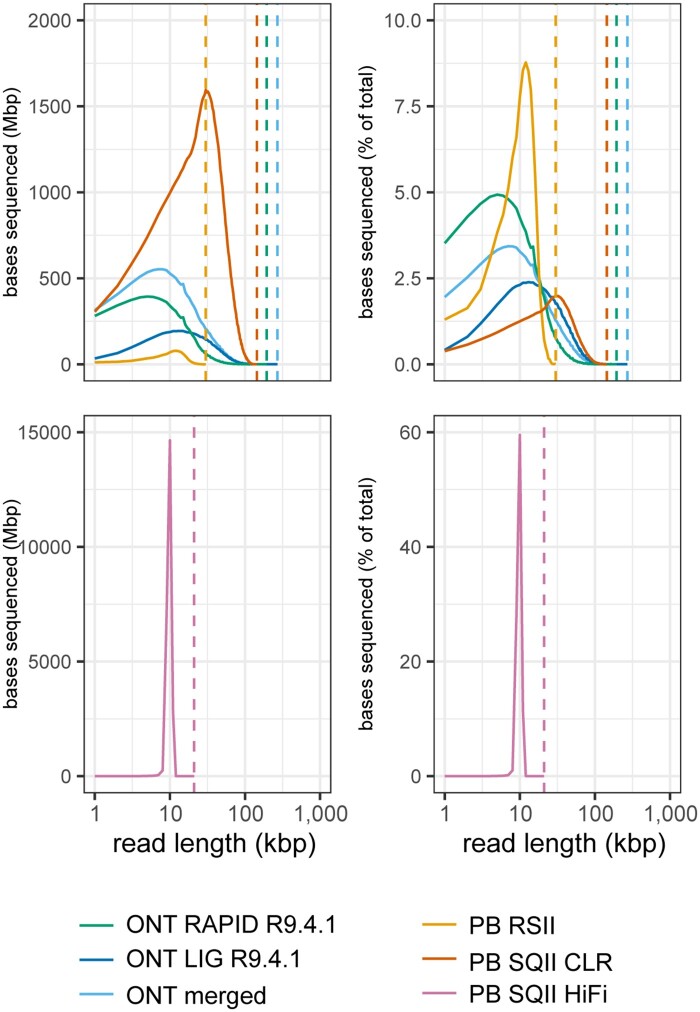
Read composition of *D. ananassae* long read libraries. Bases sequenced per read length were calculated for 1 kbp bins in each library. Reads and sequenced bases are shown as raw numbers and as percentages for complete datasets. Vertical dotted lines correspond to maximum read length for each library.

*Drosophila ananassae* sequencing data produced from PacBio and ONT libraries had read composition profiles similar to *E. coli* (Table 1; Figure 3). The PacBio CLR library had the highest read N50 value while ONT libraries had longer maximum read lengths. Together, the ONT libraries had ∼16 Gbp sequenced in “ONT pass” reads and were combined to generate *D. ananassae* assemblies (Table 1). The sequencing depth of the PacBio RS II library (∼4X) was too low to assemble individually.

**Figure 4:**
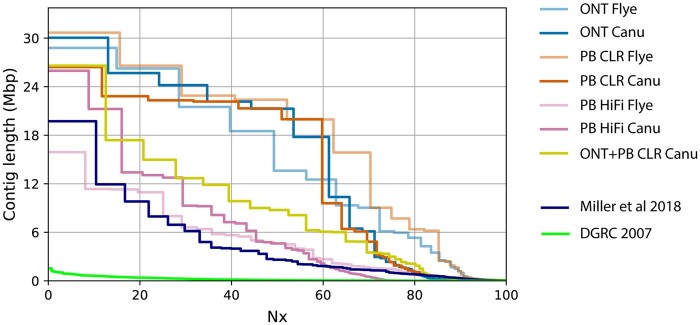
Nx plot of *D. ananassae* assemblies. Plot of Nx values for *D. ananassae* assemblies produced in this study. Each Nx value represents the shortest contig length when summed with all larger contigs totaling X% of the total assembly size. Nx values were calculated in QUAST-LG. Assemblies produced in this study were compared to contigs (broken scaffolds) of two previous assemblies of *D. ananassae* (Drosophila 12 Genomes Consortium 2007; Miller *et al.* 2018).

*Drosophila ananassae* assemblies were produced using Canu and Flye with ONT, PacBio CLR, and PacBio HiFi libraries. In addition, one hybrid Canu assembly was generated using Canu with combined read data from PacBio CLR and ONT runs. *D. ananassae* Flye assembly sizes were 193–198 Mbp and Canu assemblies were 225–295 Mbp (Table 3); the genome size estimated using flow cytometry was 212.5 Mbp (*n* = 5, SE 0.4 Mbp) and 205.4 Mbp (*n* = 4, SE 0.6 Mbp) for females and males, respectively. Assembled genome sizes are expected to be larger than the flow cytometry estimates produced here as the assemblies are composed of a complete set of autosomes, chromosome X, and chromosome Y. Flow cytometric estimates in females do not include the Y and in males include the average sex chromosome size. Assembly of ONT reads using Canu and PacBio CLR reads using Flye generated contiguous sequences >30 Mbp, and assemblies using ONT or CLR data alone were more contiguous than the hybrid assembly produced from combined datasets (Table 3; Figure 4). As expected, assemblies produced from the shorter, size-selected PacBio HiFi sequences were less contiguous (Table 3; Figure 4). PacBio HiFi assemblies were also largest compared to their CLR and ONT counterparts. Although the sequenced fly line is highly inbred, the larger HiFi assembly sizes could be in part due to an increased resolution of repetitive or heterozygous regions (Wenger *et al.* 2019; Vollger *et al.* 2020). The PacBio HiCanu assembly also likely has uncollapsed sequences; 63% of contigs (3378/5382) were constructed from one to three reads.

**Table 3:** Summary of *D. ananassae* assemblies

Library/ assembly	Assembler	Assembly size (Mbp)	Contigs	Max contig (Mbp)	Contig N50 (Mbp)	KAT completeness	Genome fraction (%)	Duplication ratio	BUSCO Complete^a^ (%)	BUSCO Duplicated (%)	Chromosome arm contigs	Chromosome arm contig size (Mbp)	Consensus identity (%)^b^	Estimated LGT
ONT (RAPID +LIG)^c^	Flye	193.0	382	28.8	13.6	98.78	87.84	1.01	98.82	0.69	24	154.7	99.901	2,581,904
ONT (RAPID +LIG)	Canu	230.4	486	30.0	21.3	98.20	94.89	1.08	98.22	1.18	15	157.6	99.843	5,325,934
PB SQ II CLR^c^	Flye	196.8	404	30.7	22.4	99.58	89.23	1.02	99.11	0.49	13	156.1	99.962	2,972,519
PB SQ II CLR	Canu	225.8	233	26.4	21.3	99.74	99.38	1.05	99.11	4.84	14	155.6	99.972	5,607,801
ONT+PB SQII CLR	Canu	211.9	228	26.6	8.8	99.66	94.28	1.04	98.82	1.97	30	156.4	99.971	5,209,858
PB SQ II HiFi^c^	Flye HiFi	197.5	482	15.9	4.6	99.53	89.98	1.01	99.21	0.49	137	156.1	99.966	2,076,708
PB SQ II HiFi	HiCanu	294.6	5382	26.0	4.6	99.75	99.16	1.36	99.31	13.43	36	160.1	99.960	10,370,226
Dana.UMIGS	NA	213.8	139	30.7	26.4	99.47	NA	NA	99.11	0.49	6	155.8	NA	5,031,104
Miller *et al.* (2018)	NA	189.2	371	19.7	2.6	99.45	84.04	1.02	99.01	0.59	87	154.7	99.886	ND
D12GC 2007^c^	NA	213.9	20,488	1.5	0.1	99.18	88.15	1.08	99.11	0.69	2278	150.6	99.893	ND

a The sum of complete and duplicated BUSCOs recovered using the arthropod odb10 dataset (1013 total).

b Mismatch and indel rates per 100 kbp were calculated by comparing the X + 2 + 3 chromosome arm contigs in Canu/Flye assemblies to the Dana.UMIGS assembly in QUAST.

c Results reported from contigs (broken scaffolds).

NA, not applicable; ND, not determined.

Since assemblies produced in this study were more contiguous than previously published *D. ananassae* genomes (Drosophila 12 Genomes Consortium 2007; Miller *et al.* 2018), a new high-quality *D. ananassae* reference genome was generated (referred to here as Dana.UMIGS) to combine the advantages of ONT/CLR read length with HiFi read accuracy. Briefly, the Dana.UMIGS assembly was constructed by combining the longest assembled contig for each of the six euchromatic chromosome arms to the PacBio CLR Canu contig set. Contig extension and merging was performed using quickmerge (Chakraborty *et al.* 2016) with donor sequences from the PacBio HiFi library. The merged assembly was polished with CLR and HiFi reads followed by manual inspection to remove contaminants and erroneously duplicated regions. The Dana.UMIGS assembly had a total size of 213.8 Mbp assembled into 139 contigs with an N50 value of 26.4 Mbp (Table 3).

### *Drosophila ananassae* assembly quality

To better understand the wide variation observed in assembly sizes across long read libraries and assemblers, assemblies were aligned to the Dana.UMIGS assembly using QUAST. The genome fraction was calculated for each assembly as the length of aligned sequence in test assemblies divided by the size of the Dana.UMIGS assembly. The genome fraction was lower in assemblies using ONT reads and Flye assemblies relative to Canu, suggesting there may be unassembled or overly-collapsed regions in these assemblies (Table 3). The duplication ratio was calculated for each assembly as the length of aligned sequence in a queried assembly divided by the length of aligned sequence in Dana.UMIGS. PacBio and Canu assemblies had higher duplication ratios, with the highest observed ratio in the HiCanu HiFi assembly (Table 3).

A reference-independent method of evaluating assembly composition was conducted using KAT (Mapleson *et al.* 2016) to determine the completeness of assembly k-mer content in comparison to k-mers present in Illumina reads generated from the same gDNA. Dana.UMIGS and the PacBio CLR/HiFi assemblies (>99%) had higher completeness scores compared to ONT (98%–99%), and PacBio Canu assemblies had higher scores compared to Flye, supporting the QUAST results (Table 3). K-mer spectra plots suggested that Canu assemblies had more duplicated content which was particularly apparent in the HiCanu HiFi assembly (Supplementary Figure S7).

The Dana.UMIGS assembly had >99% (1004/1013) arthropod BUSCOs characterized as complete (Table 3; Supplementary Table S10). Six missing BUSCO genes were also missing in previously assembled fly genomes assembled by other groups, meaning these genes may indeed be absent from *D. ananassae*. In all *D. ananassae* assemblies produced in this study, identification of complete arthropod BUSCOs was consistently high (98%–99%) (Table 3; Supplementary Table S10). As observed in *E. coli*, ONT assemblies had a higher proportion of missing and fragmented BUSCOs while PacBio assemblies had more complete BUSCOs (Supplementary Table S10).

The number of duplicated BUSCOs was also higher in PacBio assemblies compared to ONT assemblies and published *D. ananassae* genomes (Table 3; Supplementary Table S10). While there may be true duplications in *D. ananassae*, the higher duplicated BUSCOs could be a consequence of mis-assembled regions due to elevated chimeric reads as observed in *E. coli* and/or the assembly of haplotigs by Canu/HiCanu. The sequenced *D. ananassae* line was highly inbred, therefore duplicated regions are less likely due to divergence of haplotypes and more likely due to sequencing/assembly errors.

### Comparison of major chromosomes in *D. ananassae* genome assemblies

**Figure 5:**
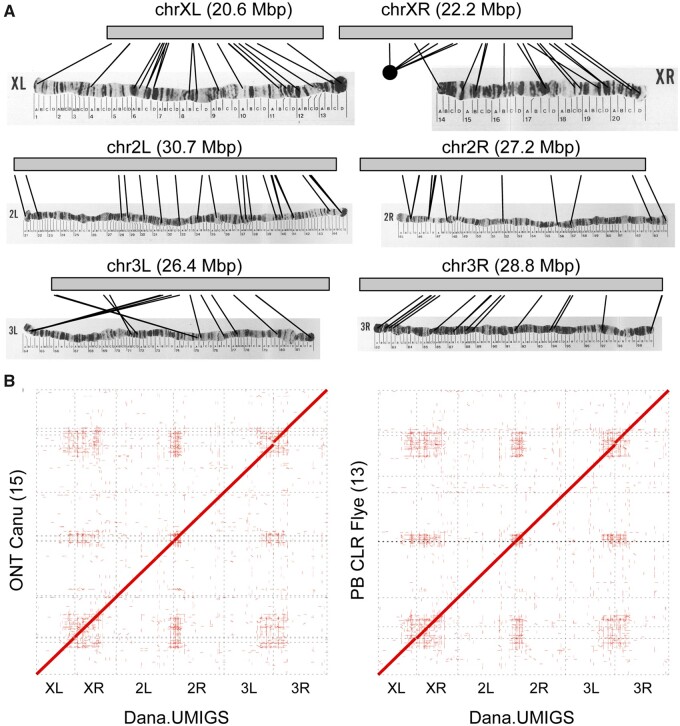
Assembly of six *D. ananassae* chromosome arms. (A) Chromosome arm contigs from the Dana.UMIGS assembly are labeled with lines connecting polytene map coordinates with estimated locus positions generated with BLAST searches. Original images for polytene maps are from (Tobari 1993). Permissions for the use of polytene map images were purchased from Karger Publishers. (B) Alignments between Dana.UMIGS chromosome arm contig and two representative test assemblies in this study (ONT Canu, PB CLR Flye). Alignments >50 kbp were identified by minimap2 and dot plots were generated using NUCmer. Numbers in parenthesis indicate the number of contigs (broken scaffolds) corresponding to chromosome arms in test assemblies.

The *D. ananassae* genome contains three metacentric euchromatic chromosomes (X, 2, 3) and two heterochromatic chromosomes (4, Y) (Hinton and Downs 1975; Schaeffer *et al.* 2008). The Dana.UMIGS genome assembly resolved the euchromatic portions of the six chromosome arms (XL, XR, 2L, 2R, 3L, 3R) into six contigs totaling 155.86 Mbp (Table 3; Figure 5A). The spatial organization of the euchromatic arms in the Dana.UMIGS assembly is supported by physical maps of polytene chromosomes, including a known strain-specific chromosomal inversion on 3L (Figure 5A; Supplementary Table S11) (Schaeffer *et al.* 2008). Chromosome arms were more highly fragmented in assemblies using individual long read libraries (Table 3; Figure 5B). Assemblies were more fragmented at the ends of chromosome arms whereas the euchromatic portions of chromosome arms were more consistently resolved in contiguous sequence (Figure 5B).

Chromosome arms from the Dana.UMIGS assembly were polished for successive rounds with PacBio CLR and HiFi data and were compared to chromosome arm contig sets from other assemblies to evaluate assembly accuracy. All assemblies had >99% consensus identity to Dana.UMIGS (Table 3). Indel frequencies in PacBio assemblies were seven to ten times lower than ONT assemblies (Supplementary Table S10). Although we did not exhaustively test genome polishing schemes for all *D. ananassae* assemblies, polishing the ONT assemblies for a single round using Illumina data reduced the frequency of mismatches and indels relative to the original assembly (Supplementary Table S10).

### Assessment of heterochromatic regions in *D. ananassae*

Repeat-rich regions are notoriously difficult to assemble and thus tend to be understated in genome reports. Substantial portions of the *D. ananassae* genome are highly heterochromatic, including all of chromosomes 4 and Y (Hinton and Downs 1975). *D. ananassae* has an expanded chromosome 4 relative to other *Drosophila* species, appearing more similar in size to chromosome X (Schaeffer *et al.* 2008; Klasson *et al.* 2014). The increased size of chromosome 4 is partially attributed to its incorporation of large lateral gene transfers (LGT) from its *Wolbachia* endosymbiont (*w*Ana) (Klasson *et al.* 2014; Dunning Hotopp and Klasson 2018). There is also evidence for retrotransposon proliferation in *D. ananassae* chromosome 4 relative to *D. melanogaster* (Leung *et al.* 2017). The total proportion of genomic heterochromatin for this *Wolbachia*-cured strain of *D. ananassae* was estimated using flow cytometry based on the proportion of thoracic underreplication (Hjelmen *et al.* 2020); females were estimated to have 20.4% late-replicating heterochromatin while males had an estimated 25.1% (replication stalling at 79.6 ± 1.6% in females and 74.9 ± 1.1% in males).

The sequencing of long reads from the cured fly line should enable the resolution of heterochromatin in fewer and longer contigs, including those containing *Wolbachia* LGT. The Dana.UMIGS assembly had 14 contigs assigned to chromosome 4 totaling 25.9 Mbp, including five contigs totaling 20.0 Mbp having alignments to chromosome 4 contigs identified in a previous *D. ananassae* genome assembly (Supplementary Figure S8) and nine contigs totaling 5.9 Mbp containing *w*Ana LGT. After sequencing Illumina libraries prepared from individual male and female flies, the median female:male sequencing depth ratio was used to assign a total of 60 Dana.UMIGS contigs, totaling 20.2 Mbp, to chromosome Y (Supplementary Figure S9). The ∼14 Mbp size difference between chromosome X and Y determined from flow cytometry estimates (2C^female^-2C^male^; 425 Mbp-411 Mbp) is smaller than the ∼23 Mbp observed size difference in contigs assigned to chromosome X versus chromosome Y, likely due to the incomplete assembly and/or annotation of chromosome Y contigs.

The amount of LGT present in each genome assembly was estimated by aligning the entire *w*Ana genome against *D. ananassae* LGT contigs using NUCmer and subsequently summing the lengths of aligned sequences using BEDtools. LGT content was primarily associated with the assembler used: Flye assemblies contained <3 Mbp of LGT, while Canu assemblies contained >5 Mbp (Table 3). ONT assemblies tended to include less LGT versus comparable PacBio assemblies with the exception of the Flye HiFi assembly (Table 3). Although the remainder of chromosome 4 and chromosome Y contigs were not assigned in all assemblies produced in this study, the larger contig sizes in ONT and PacBio CLR assemblies relative to HiFi assemblies at Nx values when x > 80 suggests that heterochromatic regions were more contiguous when assembled with longer reads (Figure 4). Lower contiguity of heterochromatic regions might be expected if sequencing technologies are biased with respect to sequencing heterochromatin or euchromatin. Reads from ONT, PacBio, and Illumina libraries were mapped to the Dana.UMIGS assembly to determine the distribution of sequencing depth across heterochromatic and euchromatic regions in the *D. ananassae* genome. The modes of sequencing depth distributions were compared to assess differences in heterochromatic versus euchromatic sequencing depth in chromosomes X, 2, and 3 in addition to LGT-containing versus non-LGT regions in chromosome 4.

**Figure 6:**
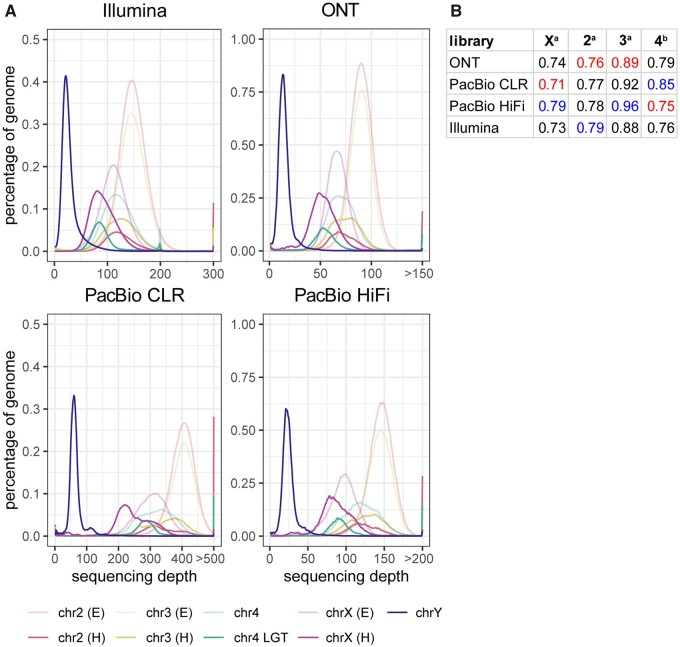
Distributions of library sequencing depth across *D. ananassae* genome. (A) Visualization of library sequencing depth in multiple *D. ananassae* genome regions. Reads were mapped to the Dana.UMIGS assembly using minimap2 (ONT/PacBio) and bwa mem (Illumina). After removing secondary alignments, sequencing depth for each library was quantified using the purge_haplotigs “hist” command. To estimate sequencing depth of chromosome Y, chromosome 4, and LGT contigs, the number of positions at each depth value were summed for all contigs assigned to those regions in the Dana.UMIGS assembly. To estimate sequencing depth of euchromatic (E) and heterochromatic (H) regions in chromosome X, 2, and 3, BAM files were subsetted with SAMTools using user-defined contig coordinates. Euchromatic regions were approximated as contig regions containing genes from the *D. ananassae* polytene map (Supplementary Table S11). Heterochromatic regions were approximated as the contig coordinates outside euchromatic intervals. The purge_haplotigs “hist” script was performed again on subsetted BAM files. Since positions having a depth value of zero consider the entirety of a contig (*e.g.*, positions with depth=0 in the chrX euchromatic BAM file is the sum of euchromatic positions with zero depth plus all heterochromatic positions), the counts of positions in each dataset with zero depth were omitted from this analysis. (B) Representation of heterochromatic read depth relative to euchromatic read depth. ^a^For chromosomes X, 2, and 3, relative representation of heterochromatic regions was calculated as mode^H^/mode^E^. ^b^For chromosome 4, relative representation of LGT regions was calculated as mode^chr4LGT^/mode^chr4nonLGT^. Red and blue values indicate the lowest and highest ratios in each column, respectively.

In all libraries, sequencing depth was lower in heterochromatic regions in chromosomes X, 2, and 3 relative to euchromatic regions (Figure 6, A and B). Five contigs assigned to chromosome 4 had a sequencing depth similar to heterochromatic regions in other fly autosomes. Nine contigs containing *w*Ana LGT had lower sequencing depth compared to chromosome 4 contigs containing no LGT (Figure 6, A and B). Distributions shapes of libraries primarily reflected the total sequencing depth of the library, although PacBio libraries had more uneven sequencing depth relative to ONT and Illumina (Figure 6A). Overall, there was no apparent read technology bias in long-read sequencing of heterochromatic regions.

### Detecting DNA modification using *D. ananassae* long read libraries

The extent of DNA methylation in *Drosophila* is not fully understood. Methylation in animals consists primarily of cytosine modification in CpG islands maintained by multiple DNA methyltransferases (DNMTs) (Goll and Bestor 2005). *D. melanogaster* lacks homologs of DNMT1 and DNMT3 but possesses DMNT2 which is highly conserved across dipterans, mouse, and human (Marhold *et al.* 2004). In the attempt to characterize methylation in *D. melanogaster*, multiple studies disagree in (1) the extent of genome-wide cytosine methylation and (2) the role of DMNT2 in this process (Lyko *et al.* 2000; Kunert *et al.* 2003; Zemach et al. 2010; Raddatz *et al.* 2013). The retention of cytosine methlylation in DNMT2-knockout embryos (Boffelli *et al.* 2014; Takayama *et al.* 2014) indicate there are unidentified methyltransferases in *Drosophila*. Global 5mC has been quantified in *D. ananassae* using liquid-chromatography-mass spectrometry (Deshmukh *et al.* 2018), but methylated motifs have not been reported.

Given its lower sequencing depth, the application of DNA modification pipelines is less reliable in *D. ananassae*. The PacBio interpulse duration (IPD) signatures of 5mC modifications are more challenging to detect relative to 6 mA and require >250X depth or enzymatic conversion of 5mC to improve detection (Pacific Biosciences 2015; Clark *et al.* 2013). Therefore, not surprisingly, we could not identify methylation signatures in *D. ananassae* using PacBio libraries. While the <40X depth in ONT libraries was too low for robust genome-wide methylation calls, the Tombo 5mC model-based calling of the ONT LIG library permitted preliminary analysis: The 1000 regions in the Dana.UMIGS assembly with the highest proportion of ONT reads supporting DNA methylation contained CG and GC dinucleotides (Supplementary Figure S10), however the precise methylation sites cannot be readily identified in more complex motifs using this method.

## Discussion

Our study demonstrates that highly contiguous assemblies can be obtained with long-read technologies and assemblies also benefit from error correction with more accurate reads. Assemblies of *E. coli* and *D. ananassae* surpassed 99.9% consensus accuracy when using long read data alone, and accuracy was further improved when using Illumina data for hybrid assembly or error correction. Circularized *E. coli* genomes can be achieved with either ONT or PacBio libraries, and the *D. ananassae* genomes assembled in this study are the most contiguous reported to date for this fly strain (Drosophila 12 Genomes Consortium 2007; Miller *et al.* 2018) with all six euchromatic chromosome arms resolved into single contigs. Additionally, DNA methylation patterns were characterized using both ONT and PacBio data. Methylation at known *E. coli* motifs was supported by both long read technologies, but the lack of reference motifs in *D. ananassae* prevented extensive analysis.

### Comparative analyses support superior performance of PacBio Sequel II libraries

PacBio Sequel II CLR sequencing represents a major advancement in sequencing throughput over previous PacBio platforms with the production of more sequencing data and longer reads versus RS II and the Sequel I (not tested here). Although ONT libraries had longer reads sequenced, Sequel II CLR libraries had a larger pool of ultra-long reads, demonstrated by higher read N50 values.

While greater sequencing depth increases the likelihood of producing high consensus accuracy resolution from error-prone reads, the subsampling of datasets confirmed that *E. coli* assemblies using PacBio sequencing were the most accurate. Highly conserved bacterial genes were also more consistently characterized in PacBio assemblies. Superior accuracy was often achieved after polishing with Illumina reads but is not advised when the underlying data used to generate the assembly is highly accurate. Polishing may not resolve all residual indel errors in homopolymer tracts, which remains a current challenge in both ONT and PacBio sequencing (Giordano *et al.* 2017; Wenger *et al.* 2019).

*D. ananassae* assemblies using ONT or CLR data alone were more contiguous than hybrid ONT-CLR assemblies. One possible explanation is that different types of sequencing errors in ONT and PacBio reads could hinder overlap detection. Although the summary statistics commonly used to describe genome assemblies (*e.g.*, contig count, contig N50, maximum contig length) were often higher in PacBio assemblies, these assemblies might also have more duplicated content from uncollapsed regions; researchers must exercise appropriate caution when choosing the “best” assembly to report. The fragmentation of *D. ananassae* assemblies generated with PacBio HiFi reads means that the choice of Sequel II sequencing mode involves a trade-off between contiguity and accuracy when sequencing large and repetitive genomes.

### PacBio sequencing is not optimal for all sequencing applications

While PacBio Sequel II CLR/HiFi demonstrated the best results for most data quality tests, there are certain situations where alternatives to PacBio sequencing might be preferred. The maximum length of ONT reads is larger such that ONT sequencing can span longer repetitive regions. In highly repetitive eukaryotic genomes a hybrid approach might be warranted; the most contiguous D. ananassae assembly was generated by combining sequences from multiple PacBio and ONT assemblies.

ONT sequencing enables a more comprehensive analysis of small sequences and DNA modifications. Our *E. coli* results demonstrated the complete omission of a 5 kbp plasmid and the underrepresentation of a 97 kbp plasmid using PacBio and ONT LIG sequencing. Conversely, ONT RAPID produced plasmid sequencing data much closer to expectedproportions. While detection of DNA methylation at adenine residues is available using both PacBio and ONT technologies, cytosine DNA methylation information can be obtained from ONT reads without additional library preparation steps. 

Chimeric reads are more common in library preparations that involve ligation. Unicycler uses long reads to generate scaffold bridges across contigs assembled with Illumina data, meaning assemblies should not be negatively impacted by chimeric reads (Wick *et al.* 2017a, 2017b). Chimeric reads may hinder assembly-free analyses such as the validation of DNA integration into eukaryotic genomes *via* LGT. Although the overall frequency of chimeric reads is low, additional investigation of the occurrence and genome-wide distribution of chimeras is needed, particularly for eukaryotic genomes.

Finally, the higher cost of entry for the PacBio platform might be prohibitive for some researchers, despite the lower cost per base for Sequel II sequencing. The ONT MinION starter kit is currently priced at $1000 USD and includes the sequencing device, one flow cell, and all other consumables necessary for the sequencing run. The cost for a PacBio Sequel II sequencing run alone (not including the device or library prep consumables) is at least twice the cost of the ONT starter kit but yields 2-5X more raw data.

### Long-read sequencing enables resolution of euchromatic genome, but challenges remain in assembling heterochromatin

The three euchromatic chromosomes in the *D. ananassae* genome were resolved into chromosome-arm length contigs when assembling ONT or PacBio reads. Increased assembly contiguity can be achieved by merging sequence sets from multiple assemblers (Chakraborty *et al.* 2016). In contrast, heterochromatic regions are more highly fragmented and appear to be underrepresented across long-read sequencing technologies. There are multiple potential explanations for the observed lower sequencing depth in *D. ananassae* heterochromatic regions. Unsuccessful mapping of long reads onto highly fragmented heterochromatic contigs and/or persistent misassembled regions in the Dana.UMIGS genome assembly could hinder accurate estimation of sequencing depth. Lower sequencing depth could also be a consequence of underreplication of heterochromatic regions previously observed in *Drosophila*. In multiple *Drosophila* tissues, stalling of S-phase prior to the completion of genome replication can leave the late-replicating regions (typically, heterochromatin) in an “underreplicated” state (Belyaeva *et al.* 1998; Johnston *et al.* 2013). Underreplication in the majority of cells in the *Drosophila* thorax (Johnston *et al.* 2013; 2020) suggests potential issues in genome assembly owing to differing sequencing depth of heterochromatic and euchromatic regions. Underreplication is widespread across the *Drosophila* phylogeny, including *D. ananassae*, and should be considered when assembling heterochromatic sequences (Hjelmen *et al.* 2020). Advances in long read sequences have enabled more consistent assembly of genomes due to its ability to span lengthy repeat regions. Additional efforts including those in the development of genome assembly algorithms are needed to account for unusual and often tissue-specific chromosome states, which may be impacting the ability to assemble heterochromatic regions of the genome.

The amount of LGT in contigs in the *D. ananassae* genome assemblies was associated with both the long read library as well as the assembly tool used. ONT assemblies contained less LGT, however this does not appear to be the result of lower heterochromatic read representation in ONT libraries. Canu-based assemblies had more LGT content in contigs, likely in part due to Canu’s algorithmic focus on resolving haplotigs. Likely in resolving haplotigs, Canu can also resolve recent duplications like *Wolbachia* LGT regions in *D. ananassae* (Klasson *et al.* 2014). In addition, the underreplicated heterochromatin may also complicate resolution of these recent duplications using sequencing depth. Manual annotation of these regions is needed for optimal characterization of *D. ananassae* LGT and the presence of true haplotypes cannot be ruled out, although this is an extensively inbred line.

### Development of new sequencing products and bioinformatics tools will continue to improve long-read sequencing

The rapid turnover of sequencing platforms and analysis pipelines will continue to improve the utility of long-read sequencing data. Since the design of this experiment, ONT have begun distribution of the R10.3 nanopore which can currently only be used with the LIG protocol but advertises to improve consensus accuracy to over 99.99%. New Sequel II sequencing chemistry released by PacBio claims to improve performance, including the reduction of baseline DNA modification scores. The increased use of long-read sequencing data has spurred a plethora of bioinformatic tools for long read overlap detection, contig assembly, and error correction (Chu *et al.* 2016; Sedlazeck *et al.* 2018; Fu *et al.* 2019). Platform-specific tools have been developed to achieve optimal results given the underlying features of long read data (*e.g.*, Arrow and Nanopolish used for polishing genomes using PacBio and ONT data, respectively). While it is possible that improved results could have been obtained in this study by using platform-specific tools, we chose tools for the study on the basis of (1) their wide usage in long read genome assembly and (2) their platform independence. Nevertheless, it is possible that the tools with the specified parameters are better able to handle the error profile of data from a specific platform, leading to the observation of superior performance in many of our tests.

## Conclusions

With the arrival of PacBio Sequel II, researchers can achieve unprecedented throughput in long-read sequencing data. The advancement of Sequel II confers an increase in consensus accuracy and a higher likelihood of sequencing across repetitive regions, although ONT sequencing might be more suitable for some sequencing applications. Increased adoption of long-read sequencing platforms promises to revolutionize genomics research.

## Funding

This work was supported by the National Institute of Allergy and Infectious Diseases, National Institutes of Health, Department of Health and Human Services [U19AI110820]; JCDH and EST are also supported by an National Institutes of Health Director’s Transformative Research Award [R01CA206188].

## Conflicts of interest

We have no competing financial interests.
